# Polyploidy Index and Its Implications for the Evolution of Polyploids

**DOI:** 10.3389/fgene.2019.00807

**Published:** 2019-09-10

**Authors:** Jinpeng Wang, Jun Qin, Pengchuan Sun, Xuelian Ma, Jigao Yu, Yuxian Li, Sangrong Sun, Tianyu Lei, Fanbo Meng, Chendan Wei, Xinyu Li, He Guo, Xiaojian Liu, Ruiyan Xia, Li Wang, Weina Ge, Xiaoming Song, Lan Zhang, Di Guo, Jinyu Wang, Shoutong Bao, Shan Jiang, Yishan Feng, Xueping Li, Andrew H. Paterson, Xiyin Wang

**Affiliations:** ^1^School of Life Sciences, North China University of Science and Technology, Tangshan, China; ^2^Center for Genomics and Computational Biology, North China University of Science and Technology, Tangshan, China; ^3^State Key Laboratory of Systematic and Evolutionary Botany, Institute of Botany, Chinese Academy of Science, Beijing, China; ^4^University of Chinese Academy of Sciences, Beijing, China; ^5^Cereal & Oil Crop Institute, Hebei Academy of Agricultural and Forestry Sciences, Shijiazhuang, China; ^6^Plant Genome Mapping Laboratory, University of Georgia, Athens, GA, United States

**Keywords:** polyploidy, angiosperm, P-index, genomics, bioinformatics

## Abstract

Polyploidy has contributed to the divergence and domestication of plants; however, estimation of the relative roles that different types of polyploidy have played during evolution has been difficult. Unbalanced and balanced gene removal was previously related to allopolyploidies and autopolyploidies, respectively. Here, to infer the types of polyploidies and evaluate their evolutionary effects, we devised a statistic, the Polyploidy-index or P-index, to characterize the degree of divergence between subgenomes of a polyploidy, to find whether there has been a balanced or unbalanced gene removal from the homoeologous regions. Based on a P-index threshold of 0.3 that distinguishes between known or previously inferred allo- or autopolyploidies, we found that 87.5% of 24 angiosperm paleo-polyploidies were likely produced by allopolyploidizations, responsible for establishment of major tribes such as Poaceae and Fabaceae, and large groups such as monocots and eudicots. These findings suggest that >99.7% of plant genomes likely derived directly from allopolyploidies, with autopolyploidies responsible for the establishment of only a few small genera, including *Glycine*, *Malus*, and *Populus*, each containing tens of species. Overall, these findings show that polyploids with high divergence between subgenomes (presumably allopolyploids) established the major plant groups, possibly through secondary contact between previously isolated populations and hybrid vigor associated with their re-joining.

## Introduction

Polyploidy is a state in which an organism or cell contains two or more basic sets of chromosomes (Van de Peer et al., 2017). An autopolyploid is formed by duplicating a genome, whereas an allopolyploid is derived from hybridization between different species with some independent evolutionary history, followed by chromosome doubling or fusion of unreduced gametes.

The prevalence of different types of plant polyploidies has long been a topic of debate (Barker et al., 2016; Soltis et al., 2016). Theoretically, autopolyploids are thought to form more frequently and involve fewer incompatibilities between the merged genomes, but allopolyploids may offer greater advantages to a new lineage due to their potential for permanent inter-genomic heterosis (Ramsey and Schemske, 2002). However, it is difficult to gauge the ratio of novel allopolyploidy to autopolyploidy in extant plants because chromosome counting often provides insufficient information to distinguish these cytotypes. A recent survey indicated that across 47 vascular plant genera, 13% and 11% of plant species could be inferred as auto- and allopolyploids, respectively (Barker et al., 2016).

Genome sequencing has provided rich evidence that polyploidy contributed substantially to the diversification of land plants (Jiao et al., 2011; Soltis et al., 2015; Kellogg, 2016; Farhat et al., 2019; Huang and Zhu, 2019; Ibiapino et al., 2019; Sessa, 2019) and crop domestication (Salman-Minkov et al., 2016; Van Drunen and Husband, 2019). Despite near-parity of occurrence of auto- and allopolyploids among extant plants, surprisingly, and interestingly, genomic analysis showed that maize (*Zea mays*), bread wheat (*Tritium aestivum*), canola (*Brassica napus*), and the common ancestor of grasses have allopolyploid origins, with some of them as young as thousands of years (canola, ∼7,500 years, and bread wheat, ∼10,000 years) (Chalhoub et al., 2014; International-Wheat-Genome-Sequencing-Consortium, 2014; Wang et al., 2015a), while others are tens of millions of years old [maize, formed ∼26 million years ago (mya) (Schnable et al., 2009; Wang et al., 2015b), and the common ancestor of grasses, ∼98 mya] (Murat et al., 2015; Wang et al., 2015b). This seems to imply that allopolyploidy may confer genetic and environmental advantages that enhance survival.

Characterization of patterns of gene deletion following the most recent whole-genome duplication in *Musa acuminata* and several other angiosperms showed two classes of polyploidy (Garsmeur et al., 2014), including “unbiased fractionation and genome equivalence” with duplicated genes deleted to an equal extent between two subgenomes and “unequal and biased fractionation” between subgenomes. The two classes were related to auto- and allopolyploidies, respectively. However, this previous analysis was based on block-by-block characterization of collinear and non-collinear genes, failing to provide a whole-genome level description of gene retention/loss. Besides, the characterization is also easily affected by post-polyploidy gene translocation, which is popular inconsideration of widespread and recurrent burst of transposon activities.

Outgroup reference genomes can often share appreciable gene orthology with polyploidy-affected genomes, providing a ready way to credibly evaluate gene loss/retention in the latter genomes. Furthermore, exploration of the frequencies of different types of polyploids and their effects on the establishment of angiosperm tribes has been difficult (Soltis et al., 2009). Therefore, by referring to well-characterized outgroup genomes, we devised novel statistics and explored patterns of gene loss between subgenomes produced by the most recent polyploidization affecting the formation of sequenced and well-assembled genomes of 44 angiosperms, and found that the level of subgenome divergence contributed to their successful expansion.

## Results

### P-Indices and Polyploidies in Angiosperm

Inferring the types of polyploidies and evaluating their evolutionary effects, we devised a statistic, the Polyploidy-index or P-index, to characterize the degree of divergence between subgenomes of a polyploid, to find whether there has been a balanced or unbalanced gene loss pattern (see *Materials and Methods* for details). When calculating P-index, a reference genome was used to show orthologous gene colinearity with the studied genome, and checking the intervening non-collinear genes would show likely gene losses in each of the inferred subgenomes produced by a paleopolyploidization event. Reciprocal gene losses in different subgenomes were reflected by the definition of P-index. Different reference genomes could be adopted; however, a well-assembled and evolutionarily close one could offer a relatively credible assessment of the studied genome.

**Figure 1:**
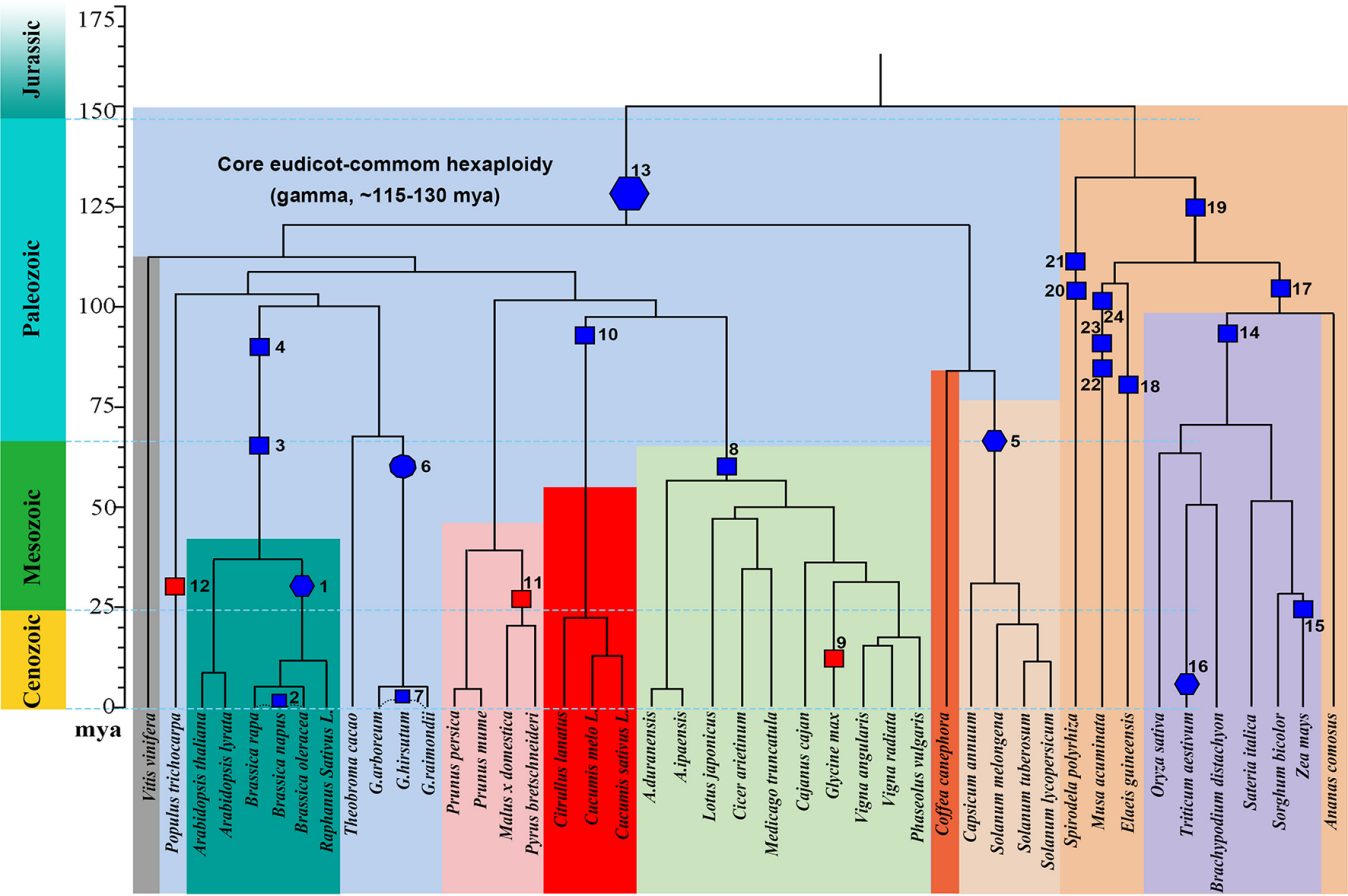
Phylogeny and polyploidies during the evolution of angiosperms. Selected sequenced angiosperms were involved, and a timescale is displayed showing dates of events. Squares are used to show tetraploidy events, and hexagons are used to show hexaploidy events. Specifically for *Gossypium*, an ancient decaploid, a decagon is used to show the polyploidy event. Filled blue polygons show inferred allopolyploidies, and red ones show autopolyploidies.

**Figure 2:**
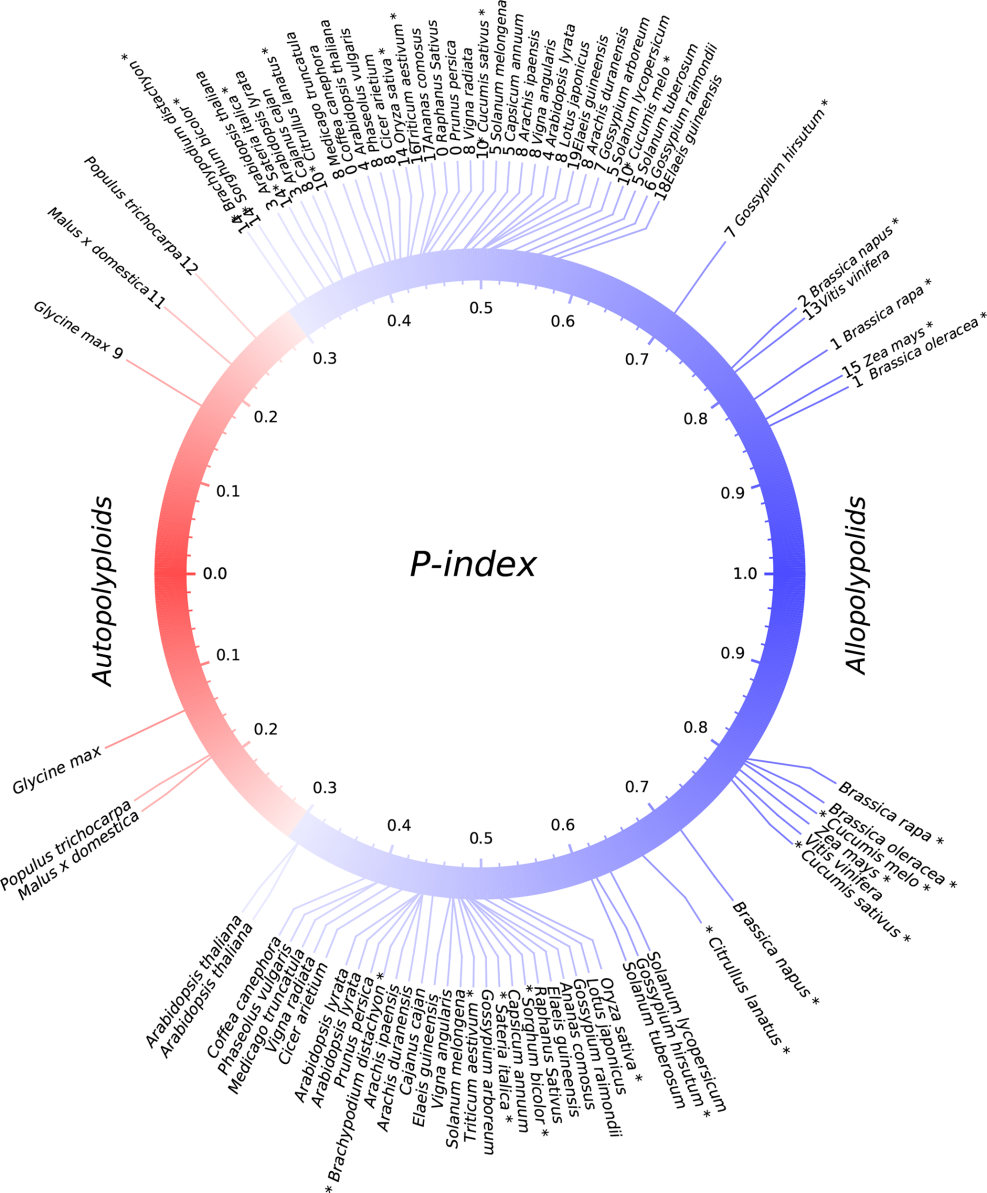
P-indices of polyploidies. The upper part shows observed P-indices for each polyploid by using the harboring genome, and the lower part shows the simulated P-index values. Known or previously inferred allopolyploids are marked with asterisks at the end of plant names. The color of the circle shows a shift from autopolyploids to allopolyploids.

We explored gene losses in 44 well-assembled angiosperm genomes affected by polyploidies (**Figure 1**) and characterized their P-indices during the evolution of these species (**Figure 2**).

### P-Indices of Known or Inferred Allopolyploidies

*Gossypium hirsutum*, *T. aestivum*, and *B. napus* are allopolyploidies in that each has two or three well diverged subgenomes, which are often based on alleles shared with related plants (**Figure 2**; **Table 1**). We checked their P-indices.

**Table 1:** P-index of polyploidy events during the evolution of angiosperms.

Plant tribe		Checked genome and polyploid event	Reference genome and polyploidy nature	P-index
**Eudicot**				
*Brassica*	1	*Brassica*-common hexaploidy	Allo	
		*B. oleracea*	*A. thaliana*	0.85
		*B. rapa*	*A. thaliana*	0.82
	2	*B. napus*-specific tetraploid	Allo	
		*B. napus*	*B. rapa*	0.78
		*B. napus*	*B. oleracea*	0.76
*Arabidopsis*	3	Arabidopsis tetraploid 1		
		*A. thaliana*	*V. vinifera*	0.34
		*A. lyrata*	*V. vinifera*	0.36
	4	Arabidopsis tetraploid 2		
		*A. thaliana*	*V. vinifera*	0.41
		*A. lyrata*	*V. vinifera*	0.50
Solanaceae	5	Solanaceae-common hexaploidy	Allo	
		*S. lycopersicum*	*C. canephora*	0.53
		*S. tuberosum*	*C. canephora*	0.56
		*Capsicum annuum*	*C. canephora*	0.50
		*Capsicum annuum*	*V. vinifera*	0.48
		*S. melongena*	*C. canephora*	0.47
Gossypodium	6	Gossypodium-common decaploidy	Allo	
		*G. raimondii*	*T. cacao*	0.47
		*G. raimondii*	*V. vinifera*	0.57
		*G. arboreum*	*T. cacao*	0.42
		*G. arboreum*	*V. vinifera*	0.51
	7	*G. tetraploidy*	*Allo*	
		*G. hirsutum*	*G. raimondii*	0.70
Fabaceae	8	Fabaceae-common tetraploidy	Allo	
		*M. truncatula*	*V. vinifera*	0.39
		*V. radiata*	*V. vinifera*	0.45
		*V. angularis*	*V. vinifera*	0.49
		*C. arietium*	*V. vinifera*	0.42
		*P. vulgaris*	*V. vinifera*	0.42
		*L. japonicus*	*V. vinifera*	0.50
		*C. cajan*	*V. vinifera*	0.36
		*A. ipaensis*	*V. vinifera*	0.48
		*A. duranensis*	*V. vinifera*	0.51
	9	Soybean-specific tetraploidy	Auto	
		*G. max*	M. *truncatula*	0.17
Cucurbitaceae	10	Cucurbitaceae-common tetraploidy	Allo	
		*C. lanatus*	*V. vinifera*	0.38
		*C. sativus L*.	*V. vinifera*	0.56
		*C. melo L*.	*V. vinifera*	0.67
*Malus*	11	Apple-specific autotetraploidy	Auto	
		*M. domestica*	*P. persica*	0.22
		*M. domestica*	*P. mume*	0.22
*Populus*	12	Poplar-specific autotetraploidy	Auto	
		*P. trichocarpa*	*P. persica*	0.26
Major eudicot	13	Major eudicot-common hexaploidy	Allo	
		*V. vinifera*	*V. vinifera*	0.79
**Monocot**				
Poaceae	14	Grass-common tetraploidy	Allo	
		*O. sativa*	*A. comosus*	0.43
		*S. bicolor*	*A. comosus*	0.41
		*S. italica*	*A. comosus*	0.44
		*B. distachyon*	*A. comosus*	0.39
	15	*Zea*-specific tetraploidy	Allo	
		*Z. mays*	*O. sativa*	0.84
		*Z. mays*	*S. italica*	0.69
		*Z. mays*	*S. bicolor*	0.71
	16	*Triticum*-specific hexaploidy	Allo	
		*T. aestivum*	*A. tauschii*	0.42
		*T. aestivum*	*T. urartu*	0.43
		*T. aestivum- 1*	*H. vulgare*	0.75
		*T. aestivum*-2	H. vulgare	0.52
*Ananas*	17	*Ananas* tetraploidy	Allo	
		*A. comosus*	*E. guineensis*	0.39
		*A. comosus*	*S. polyrhiza*	0.44
		*A. comosus*	*V. vinifera*	0.42
*Elaeis*	18	*Elaeis*-specific tetraploidy	Allo	
		*E. guineensis*	*A. comosus*	0.58
		*E. guineensis*	*S. polyrhiza*	0.50
	19	Commelinids-common tetraploidy	Allo	
		*E. guineensis*	*S. polyrhiza*	0.50
*Spirodela*	20	*S. polyrhiza* tetraploidy 1	Allo	
		*S. polyrhiza*	*V. vinifera*	0.47
	21	*S. polyrhiza* tetraploidy 2	Allo	
		*S. polyrhiza*	*V. vinifera*	0.44
*Musa*	22	*Musa* tetraploidy 1	Allo	
		*M. acuminata*	*E. guineensis*	0.37
	23	*Musa* tetraploidy 2	Allo	
		*M. acuminata*	*E. guineensis*	0.35
	24	*M*. tetraploidy 3	Allo	
		*M. acuminata*	*E. guineensis*	0.44

*TG. hirsutum* has a genotype AADD, or two sets of subgenomes, with AA from *G. arboreum* (Li et al., 2014) and DD from *Gossypium raimondii* (Paterson et al., 2012). Here, we used *G. raimondii* as the reference, and estimated the P-index of G. *hirsutum* to be 0.70. *B. napus* (genome AACC) was formed by recent allopolyploidy between ancestors of *B. rapa* (Asian cabbage or turnip, genome AA) and *B. oleracea* (Mediterranean cabbage, genome CC). With the two progenitor genomes as reference, respectively, we inferred that the P-indices of *B. napus* to be 0.76 and 0.78. Although the two progenitor genomes cannot be unequivocally traced within the genome of modern *Z. mays* (maize), there was a number of evidence that maize arose as an allotetraploid (Swigonova et al., 2004; Schnable et al., 2011; Renny-Byfield et al., 2017). Here, with *Sorghum bicolor* as the reference, we estimated maize P-index to be 0.71.Using *Sateria italica* (foxtail millet) as the reference, the P-index is inferred to be 0.69. The grass-common tetraploid ancestor was inferred to be an allotetraploid based on incongruent repetitive element accumulation (Murat et al., 2015). Here, we inferred its P-index to be 0.39–0.44, respectively, by using *Oryza sativa*, *S. bicolor*, *S. italica*, and *Brachypodium distachyon* as the reference.

A little modification to the above estimation of P-index can accommodate the situation of paleo-hexaploidy or whole-genome triplication. Wheat has a genotype AABBDD, which arose as a result of two polyploidization events. The first of these is estimated to have occurred several hundred thousand years ago and brought together the genomes of a diploid related to the wild species *Triticum urartu* (2n = 2x = 14; AA; 2n is the number of chromosomes in each somatic cell and 2x is the basic chromosome number) and a species from the Sitopsis section of *Triticum* that is believed to be related to *Aegilops speltoides* (2n = 14; SS) (Petersen et al., 2006). This hybridization formed the allotetraploid *Triticum turgidum* (2n = 4x = 28; AABB), an ancestor of wild emmer wheat cultivated in the Middle East and *T. turgidumsp*. *durum* grown for pasta today. A second hybridization event between *T. turgidum* and a diploid grass species, *Aegilops tauschii* (DD), produced the ancestral allohexaploid *T. aestivum* (2n = 6x = 42, AABBDD) (Nesbitt and Samuel, 1996; Petersen et al., 2006). Here, with *A. tauschii* and *T. urartu* as the references, respectively, the bread wheat P-index was inferred to be ∼0.42; with *H. vulgare* (barley) as the references, the P-index was inferred to be ∼0.75 of the first hybridization event that formed *T. turgidum*, and the P-index was inferred to be ∼0.52 of the second hybridization event that formed *T. aestivum*.

### P-Indices of Inferred Autopolyploidies

Previously, comparative genomic analysis indicated balanced gene retention/loss between duplicated regions and proposed that *Glycine max*, *Populus trichocarpa*, and *Actinidia chinensis* have autotetraploid ancestors (Liu et al., 2017; Wang et al., 2017; Zhao et al., 2017; Wang et al., 2018). Interestingly, we found that each of them has a rather small P-index (*G. max*: 0.17, *P. trichocarpa*: 0.26) (**Figure 2**; **Table 1**).

### Summarization of P-Index in Angiosperms

We summarize below our inferences from the P-index analysis about polyploidization events during the evolution of angiosperms. The events are numbered, and the descriptions below will follow their numbered order.

The Brassicas likely share a hexaploid ancestor (Polyploidy event 1 in **Figure 1**, with P-index = 0.82 or 0.85), inferred based on *Brassica rapa* and *Brassica oleracea* genomes, respectively, using *Arabidopsis thaliana* as reference. The known allotetraploid (Polyploidy 2) *B. napus* has a P-index 0.76 or 0.78, respectively, inferred with *B. rapa* and *B. oleracea* as references. Here, different combinations of checked genomes and references yielded similar indices.

Arabidopsis was affected by recursive polyploidies, one hexaploidy shared with major eudicots and two tetraploidies shared with Brassicas (Polyploidies 3 and 4). For the latter two events, named β and α temporally (Bowers et al., 2003), we aligned the Arabidopsis homoeologous regions with the *Vitis vinifera* genome, and by considering sequence similarity between them, we deduced homoeologous regions produced by each event. We found both events to have a P-index ∼0.35.

The Solanaceae plants share a hexaploid ancestor (Polyploidy 5) with a P-index = 0.53 and 0.56, respectively, inferred based on *Solanum lycopersicum* (tomato) and *Solanum tuberosum* (potato) genomes, using *Coffea canephora* (coffee) as an outgroup reference. There are three sets of homoeologous regions, so we calculated P-indices considering gene retention differences between any two of them (see *Materials and Methods* for details).

*Gossypium* (cotton) species share a decaploid ancestor (Polyploidy 6), with P-indices of 0.47 or 0.57, inferred with *G. raimondii* by referring to *Theobroma cacao* and *V. vinifera*, respectively. Being a decaploid (Polyploidy 7) with five sets of homoeologous regions, as for hexaploids, we calculated P-indices considering gene retention differences between any two homoeologous regions (see *Materials and Methods* for details). Taking the P-index as a kind of distance, for each reference chromosome from *V. vinifera* and *T. cacao*, we found that two homoeologous regions often group together, with an integrated P-index of 0.23 and 0.28, and the other three sets group together, with a P-index of ∼0.44 and 0.43, and between the two groups, the P-index is 0.72 and 0.56.

Fabaceae plants share a tetraploid ancestor (Polyploidy 8), with P-indices of 0.39–0.53, inferred with various legumes, including *Medicago truncatula*, *Cicer arietium*, *Lotus japonicus*, *Arachis duranensis*, *Arachis ipaensis*, and *Vigna radiata*, by referring to outgroups *V. vinifera* and *C. canephora*, respectively. These variations in the P-index do not affect classification of the polyploid legume ancestor as a tetraploid and can be largely attributed to the different assembly levels of these genomes. The more recent soybean-specific duplication (Polyploidy 9) is of a P-index = 0.17.

Cucurbitaceae plants share a tetraploid ancestor (Polyploidy 10), with P-indices of 0.38–0.67, inferred using *V. vinifera* as reference. As with *Malus x domestica*, inference of P-indices based on *Prunus persica* and *Prunus mume* came to the estimation of P-index values for their shared polyploidization event (Polyploidy 11, P-index = 0.22). An analysis of poplar genome inferred a P-index = 0.25 (Polyploidy 12) for the Salix-common tetraploidization event, using *P. persica* as reference.

We checked the P-index of the major-eudicot common hexaploidization (Polyploidy 13). Based on inference with the *V. vinifera* genome, which has preserved much of the genome structure of the common ancestor, and by devising a modified approach to exploit triple chromosomal homoeology in the genome, we inferred the P-index (0.79) without a well-assembled outgroup reference genome.

For grasses, a comparative analysis of *O. sativa*, *S. bicolor*, *Setaria italica*, and *B. distachyon* homoeologous regions using *Ananas comosus* as an outgroup reference inferred the grass-common-tetraploid ancestor to have P-indices of 039–0.44 (Polyploidy 14). This is much smaller than the P-index of the lineage-specific maize polyploidy (Polyploidy 15 with P-indices = 0.69-0.84), inferred respectively, with *O. sativa*, *S. bicolor*, and *S. italica* as outgroup references, but similar to the P-index of allohexaploid wheat (Polyploidy 16 with P-index = 0.42/0.43).

Grasses and *A. comosus* share a polyploid ancestor (Polyploidy 17), and comparing the *A. comosus* genome to references *Elaeis guineensis*, *Spirodela polyrhiza*, and *V. vinifera*, we obtained P-indices of 0.39–0.44.

*E. guineensis* was affected by two polyploidies (Polyploidies 18 and 19), one being lineage specific and the other being common to commelinid plants, including grasses and *A. comosus*. The Elaeis-specific polyploidy was checked by referring to *A. comosus* and *S. polyrhiza*, respectively, and yielded a P-index ≥0.50, and the commelinid-common polyploidy was checked by referring to *S. polyrhiza* and also yielded a P-index of 0.50. *S. polyrhiza* was affected by two tetraploidies (Polyploidies 20 and 21), and both events were checked by referring to *V. vinifera* to find P-indices of 0.44 and 0.47, respectively. *Musa acuminata* was affected by three sequential tetraploidies (Polyploidies 22–24), and they were checked by referring to *E. guineensis* to find P-indices of 0.35, 0.37, and 0.44, respectively.

### Allopolyploids are the Major Type

By checking the situations in known or previously inferred allo- or autopolyploidies, as summarized above, we found that 87.5% of 24 paleopolyploid events during angiosperm evolution were likely allopolyploids, while autopolyploids was only a small part of it. Grossly, as to the above summary, we found that allopolyploids were responsible for the establishment of major land tribes such as Poaceae, Fabaceae, Solanaceae, and Brassicaceae, and large groups such as monocots and eudicots. By contrast, autopolyploidies were likely responsible for only a few small genera, including Glycine (soybean), Malus (apple), and Populus (poplar).

## Discussion

While providing no absolute division between allo- and autopolyploidies, we consider that P-indices > 0.3, including those in *B. napus*, *B. oleracea*, *Z. mays*, and *G. hirsutum*, seem more likely to be paleo-allopolyploidies and smaller values to be paleo-autopolyploidies. Whether or not a qualitative distinction (allo- versus autopolyploid) can be made at this precise P-index threshold, the degree of divergence between subgenomes of a polyploid remains an informative parameter. Extending this criteria to other plants considered here suggests that the majority (>87%) of paleopolyploidization events are likely paleo-allopolyploidies. These include events occurring during the ∼150-million-year history of angiosperms and resulting in large plant groups, such as a hexaploidy contributing to the establishment of major eudicots (Bowers et al., 2003) and polyploidizations contributing to the establishment of the largest plant families (Soltis et al., 2005), including the third and fifth largest angiosperm families [Fabaceae (∼19,000 species) (Young and Bharti, 2012) and Poaceae (∼12,000 species) (Grass-Phylogeny-Working-Group, 2001)], as well as two economically important families [Brassicaceae (∼4,000 species) and Solanaceae (∼2,700 species)] (Chase and Fay, 2001; Magallon and Sanderson, 2001; Bremer et al., 2003), respectively. Another well-represented plant family, Rosaceae, lacked family-common polyploidy events and is relatively small (∼3,000 species), similar to Solanaceae. In contrast, likely autopolyploidies as described recently with genomic data (Liu et al., 2017; Wang et al., 2017), contributed only to the establishment of very small tribes, such as the *Malus*, *Populus*, and *Glycine* genera, composed of 20–50 species, respectively. Indeed, these three genera were not even among the largest in their respective plant families.

Overall, these findings result in an estimate of >99.7% of angiosperms that have genomes likely derived directly from allopolyploidization, which might have influenced their biological functions and potential for evolutionary success. This suggests important advantages of allopolyploidies over long evolutionary timescales, at least one of which may be linked to hybrid vigor (Chen, 2010). Stebbins proposed that polyploids may arise through secondary contact between two populations isolated and diverging somewhat (Stebbins, 1985). Although he provided little details, the present findings through genomics analysis support his prudent hypothesis in that after some divergence, two populations could rejoin to produce allopolyploids with the vigor to survive, and even prevail over long evolutionary time.

The present findings imply that when polyploidy has two similar genomes (likely autopolyploids), their divergence and expansion may be inherently restricted. The extent of restriction may be related to the similarity or even identity among subgenomes inherited from two parents with likely the same or highly similar backgrounds in sequence, genetics, and epigenetics. Although large-scale asymmetric gene losses may restore diploid (disomic) heredity, symmetric gene losses in autopolyploids may restrict genetic innovation by allowing continuing polysomic inheritance. Indeed, allopolyploid gene loss may not only be asymmetric but complementary, creating interdependence between subgenomes and perhaps creating more opportunities for reweaving genetic and epigenetic elements to build novel regulatory pathways and networks (Comai, 2000; Chen, 2010; Sattler et al., 2016).

Very recently, it was proposed that polyploid species were more likely to be domesticated than their wild relatives, especially monocots in which 54% of crops are polyploids versus 40% of wild species, suggesting that polyploidy conferred genetic predisposition for successful domestication (Salman-Minkov et al., 2016). Here, we have shown that plants with more diverged (presumably allo-) subgenomes are more likely to survive over long evolutionary timescales than plants with less diverged (presumably auto-) subgenomes. Integrating these findings, natural allopolyploidies may be more likely than autopolyploidies to be involved in domestication. Although the plants considered here were often allopolyploids formed millions of years ago, they still may have had genetic preconditions such as strong hybrid vigor that favored domestication.

Among the eudicot plants characterized, after the shared major-eudicot-common hexaploidy, there are five sequenced plants not affected by further polyploidy, including *V. vinifera*, *T. cacao* (Malvaceae), *P. persica*, *P. mume*, and *C. canephora* (Rubiaceae), which are all woody plants. Comparatively, all the sequenced eudicot plants affected by further polyploidy, excepting *Malus* and *Pyrus* lineages affected by an autopolyploidy, are herbaceous plants. Although the plant families involved often have both woody and herbaceous plants, and the plants studied here cannot constitute a sound sample, it is an intriguing hypothesis for further investigation that ancestral woody plants may be more likely to avoid further polyploidies, perhaps having acquired a stronger capability due to hybrid vigor to withstand harsh environmental changes.

Several findings indicate the robustness of P-indices for “diagnosing” the nature of polyploidies, which is supported by including several known or inferred allo-/autopolyploids in the analysis. Firstly, known and previously inferred allopolyploidies always had larger P-indices and often grouped together. These include *B. napus*, *B. rapa*, *B. oleracea*, *G. hirsutum*, and *T. aestivum*. The pan-grass tetraploid was suggested to be an allotetraploid, and we found P-index values grouping it with known allopolyploids. *Z. mays* was reported to have two extensively diverged genomes, and here it was inferred to be an allotetraploid with a large P-index.

Analysis of different species that shared common polyploidy events resulted in similar conclusions. For example, we adopted *O. sativa*, *S. bicolor*, and *S. italica* as outgroups to evaluate gene loss in maize subgenomes. These different evaluations yielded similar P-index values. Further, very similar P-indices were inferred in multiple evaluations, involving *B. napus*, *G. max*, *M. truncatula*, *A. comosus*, and others, while adopting different references. Indeed, different references provided opportunities to perform random sampling of lost or retained genes in the genomes evaluated, which supported the robustness and effectiveness of the P-index in a statistical sense.

Computational simulation indicates that the P-index is a robust measurement of gene retention differences between homoeologous chromosomal regions (**Figure 2**). By considering gene loss rules revealed previously (Wang et al., 2016), we mimicked the occurrences of auto- or allopolyploidies by artificially constructing subgenome sequences for each plant considered and then simulated gene losses. Eventually, we calculated the simulated P-indices and found that they were quite similar to observed ones (**Figure 2**). For those with observed P-indices <0.3, related to the predicted autopolyploidies, if assuming a balanced gene loss between subgenomes, the simulated ones are also < 0.3, and vice versa. This shows the robustness of the P-index measurement of gene retention/loss in subgenomes produced by polyploidies, and is useful to infer the nature of polyploidies. This also shows that near-geometrical random loss of continual runs of genes is a good description of genomic fractionation after polyploidies.

In synthesis, paleo-allopolyploidies may have contributed much to the divergence and establishment of major and large angiosperm—even millions of years after their formation, allopolyploid genomes may still have a predisposition for speciation and domestication. These capabilities may be related to “intergenomic hybrid vigor” produced by merging two divergent genomes with histories of adaptation to different ecological niches. Hybrid vigor has been extensively explored by breeders, to produce new crops to increase their yields and/or quality. Comparatively, autopolyploidies have genomic drawbacks in evolution, restricting genetic diversity and evolutionary divergence.

## Materials and Methods

### Materials

*G. max* and *M. truncatula* genomes and their gene annotations were downloaded from JGI, version 2.0, and https://phytozome.jgi.doe.gov/pz/portal.html, version 4.0, respectively (Schmutz et al., 2010; Schmutz et al., 2014). The other plant genomes and annotations were also downloaded from public databases (**Supplementary Table 1**).

### Gene Colinearity Inference

With annotated genes as input, chromosomes from within a genome or between different genomes were compared. First, by performing BLASTP (Altschul et al., 1990), protein sequences were searched against one another to find potentially homologous genes (E-value = 1 × 10^−5^, Score > 100). A higher E-value may involve more-diverged homologous genes, and thus gene colinearity, describing a batch of genes preserving ancestral gene order, would then complement this loose requirement of gene similarity to help identify very old evolutionary events rather than jeopardize the effort here. Second, the information about homologous genes was used as input for the software ColinearScan and MCSCAN (Wang et al., 2006; Wang et al., 2012) to locate homologous gene pairs in colinearity and to perform pairwise alignment of chromosomal segments using collinear genes as anchors. The key parameter, the maximum gap between neighboring genes along a chromosome sequence in colinearity with genes along the counterpart chromosome sequence, was set to be 50 intervening genes, which was proven to be successful in previous genomics research (Wang et al., 2015b; Wang et al., 2016). Finally, a polyploidy-affected genome and its reference genome were aligned. The reference genome must have avoided the polyploidization event, and the closest relative(s) that had been sequenced were used as references. Concisely, a whole-genome duplication would produce two subgenomes, each containing chromosomes that broke afterwards. Here, the reference genome was used to pitch the broken segments of a chromosome together, eventually to reconstruct the ancestral chromosomes. The chromosomes with higher gene losses were inferred to be from a sensitive subgenome, whereas the others from a dominant subgenome (Schnable et al., 2011). If the reconstructed chromosomes show no difference in gene loss, they were assigned arbitrarily to each subgenome. Details of the implementation and usage of software to perform multiple genomic alignment and inference of gene paralogy and orthology, and characterization of polyploidies, can be found in our previous publications in grasses (Wang et al., 2015b), and comparative analysis of *Gossypium*, *T. cacao*, and *V. vinifera* (Wang et al., 2016).

### Outgroup-Supported Statistics to Measure Subgenome Difference

We devised a statistical approach to quantify fluctuations of gene retention differences, aiming at providing a mathematical method to evaluate the similarity between homoeologous chromosomes in polyploids. The duplicated (or homoeologous) sequences of a considered polyploidy-affected genome were mapped onto a selected reference genome, which avoided the polyploidization event. Supposing that there were *K* chromosomes in the referenced genome, the subgenomes *A* and *B* identified in the considered genome, no matter whether there is one being dominant or not, let us divide each pair of homoeologous chromosomes into *N_c_* windows, each with *M* (such as 100) genes. For the *i*th window of a specified homoeologous chromosome pair, we have gene retention rates *A_i_* and *B_i_* relative to the reference genome, conferring a “polyploid gene loss index” of:

$$p-index=\frac{1}{K}\sum_{c=1}W_{c}abs\left[\frac{\sum_{i=1}^{N_{c}}\frac{A_{i}-B_{i}}{abs\left(A_{i}-B_{i}\right)}\times \delta_{i}}{N_{c}-\delta \left(N_{c}\right)}\right],$$

which has a value between 0 and 1. Gene retention is supported by gene colinearity between the referenced genome and at least one of the subgenomes. Shorter chromosomes or referenced chromosomes preserving fewer collinear genes may lead to more volatility; therefore, weight for a chromosome is evaluated with:

$$W_{c}=\frac{N_{c}}{\sum_{i=1}N_{i}}\;\left(c=1,2,\cdots ,K\right).$$

We remove sliding windows having highly similar retention rates by defining the evaluating coefficient as:

$$\delta_{i}=\{\begin{array}{c} 0,ifd_{i}<\;0.05\\ 1,ifd_{i}\geq \;0.05 \end{array};$$

in which gene retention difference level is defined as:

$$d_{i}=abs\left(A_{i}-B_{i}\right)/\left[\left(A_{i}+B_{i}\right)\times 0.5\right];$$

and δ (*N_c_*) denotes the number of windows with δ_i_ = 0.

We explored several different definitions of the P-index (see methods for details), and although values shifted, we obtained similar results as shown below.

In that small gene loss difference in certain regions may contribute much to the index, we then define the gene retention difference level as:

$$d_{i}=abs(A_{i}-B_{i})/[(A_{i}+B_{i})\times 0.5];$$

we remove those regions by defining the evaluating coefficient as:

$$\delta_{i}=\left\{ \begin{array}{l} 0,ifd_{i}<0.05\\ 1,ifd_{i}\geq 0.05 \end{array}\right.;$$

and then use δ (*N_c_*) to denote the number of windows with δ_i_=0. Eventually, we define the polyploid gene loss index as:

$$P\text{-}index=\frac{1}{K}\sum_{c=1}W_{c}abs\left[\frac{\sum_{i=1}^{N_{c}}\frac{A_{i}-B_{i}}{abs(A_{i}-B_{i})}\times \delta_{i}}{N_{c}-\delta (N_{c})}\right],$$

where we define

$$W_{c}=\frac{N_{c}-\delta (N_{c})}{\sum_{i=1}N_{i}-\delta (N_{i})}\;....(c=1,2,...,K).$$

For polyploids that have multiple sets of homoeologous chromosomes, that is, the number of subgenomes, *S* > 2, the above P-index formulas can be transformed into:

$$p\text{-}index=\frac{1}{K}\sum_{c=1}W_{c}abs\left[\frac{\sum_{\left(k<j\right)}\sum_{i=1}^{N}\left[\frac{A_{ki}-B_{ji}}{abs\left(A_{ki}-B_{ji}\right)}\right]\times \delta_{i}}{N\times C_{s}^{2}-\delta \left(N\right)}\right],$$

where the evaluating coefficient δ_i_ is redefined as:

$$\delta_{i}=\left\{ \begin{array}{l} 0,if\sum_{\left(k,j\right)}\left[\left(A_{ki}-B_{ji}\right)/\left(A_{ki}+B_{ji}\right)\times 0.5\right]\times \left[1/S\right]<0.05\\ 1,if\sum_{\left(k,j\right)}\left[\left(A_{ki}-B_{ji}\right)/\left(A_{ki}+B_{ji}\right)\times 0.5\right]\times \left[1/S\right]\geq 0.05 \end{array}\right..$$

δ (N) is defined as the number of sliding windows with δ_i_ = 0.

### Calculation of the P-Index of a Polyploidy Affecting Grape as the Reference

Thanks to its conservative genome structure, the *V. vinifera* genome revealed a hexaploid ancestor of major eudicots. To calculate its P-index, we adopted a different method from the calculations with a reference genome. Here, we aligned the triple homoeologous regions. Theoretically, a specific *V. vinifera* genomic region will actually have two homoeologous regions. By referring to this specific region, and comparing the two homoeologous regions to it, we found gene retention and loss in these homoeologs. If an ancestral gene had been lost in the specific referring region, the information of its retention/loss was overlooked here. In that most gene losses after polyploidy occur randomly, this is equivalent to making a sampling experiment. With the above information on gene retention and loss, we calculated the P-index similar to when a reference genome was available.

Although the grape genome is the least fragmented, it could not be adopted to analyze all the other eudicot plants. The divergence levels of different families and genera from grape are rather different. Some relatively divergent plants would have rather fragmented orthologous chromosomal regions with grape, and have rather different gene contents due to gene translocation, duplication, new gene formation, or pseudogenization. This led to difficulty in reconstructing ancestral chromosomes and subgenomes, and small numbers of collinear genes, affecting the credibility of inferred P-index. Therefore, if possible, a relatively close and well-assembled genome should be adopted to evaluate the gene loss balance in subgenomes of the studied plant affected by polyploidization.

### Calculation of the P-Index of Multiple Polyploidies Affecting the Same Genome

Some genomes have been affected by multiple polyploidies, such as Arabidopsis, *S. polyrhiza*, and Musa, and no intervening reference genome has been available to provide better dissection. Here, we describe how we calculated the P-indices for the multiple polyploidies affecting the same genome with Arabidopsis as an example. Arabidopsis was affected by recursive polyploidies, with one being shared with major eudicots and the other two being tetraploidies shared with brassicas. For the latter two events, named beta and alpha, temporally, as previously reported, we checked by using *V. vinifera* as a reference. We aligned the Arabidopsis homoeologous regions with the *V. vinifera* genome, and by considering the sequence similarity between them, we were able to identify homoeologous regions produced by each event. Theoretically, a *V. vinifera* genomic region will have four orthologous/homoeologous Arabidopsis regions, alpha11, alpha12, alpha21, and alpha22, with the former two corresponding to beta1 and the latter two corresponding to beta2. To find the P-index of the alpha polyploidy, we counted retained and lost genes by comparing alpha11 and alpha12 to the reference, and by comparing alpha21 and alpha22 to the reference. To find the P-index of the beta polyploidy, we counted retained and lost genes by comparing each of (alpha11, alpha12) and each of (alpha21, alpha22) in a combinational manner, and the P-index formula for multiple subgenomes was implemented.

### Computational Simulation of the P-Index

Our previous research indicated that genes were often lost in continual runs following a near-geometrical random distribution (Wang et al., 2016). Here, we characterized the observed distribution of gene losses along chromosomes and performed a random experiment by deleting genes in runs in the respective reference genomes. We simulated balanced or unbalanced gene losses to predict P-indices, to see whether they are similar to the observed P-indices for a considered polyploidy event, being considered auto- or allopolyploidy. For polyploidies with P-indices > 0.3, two different gene loss distributions were generated, and they were used to generate two pseudo-subgenomes, which were compared with the reference genome to calculate a P-index. For those polyploidies with P-indices ≤ 0.3, the same gene loss distributions were generated, and also two sets of pseudo-subgenomes were generated to calculate a P-index. For genomes with multiple subgenomes, multiple gene loss distributions and corresponding pseudo-subgenomes were generated to infer a P-index.

## Data Availability

The raw data supporting the conclusions of this manuscript will be made available by the authors, without undue reservation, to any qualified researcher.

## Ethics Statement

No human studies are presented in this manuscript. No animal studies are presented in this manuscript. No potentially identifiable human images or data is presented in this study.

## Author Contributions

XW, JPW, and JQ conceived and supervised this project. PS, XM, JY, YL, SS, TL, FM, CW, XL, HG, XJL, RX, JYW, SB, SJ, SJ, YF, and XPL performed the formal analysis. JQ, LW, WG, XS, LZ, and DG contributed to the method. PS, YL, TL, and JY contributed to the software. JYW, SB, SJ, YF, and XYL collected and handled the data. JPW and JQ wrote the original draft. XW and AP reviewed and edited the draft. XW supervised the whole work.

## Funding

We appreciate financial support from the Ministry of Science and Technology of the People’s Republic of China (2016YFD0101001), the National Natural Science Foundation of China (31371282 to XW, 31510333 to JPW, and 31661143009 to XW), the National Natural Science Foundation of Hebei Province (C2015209069 to JPW and C2014209201 to LW), and the Tangshan Key Laboratory Project to XW.

## Conflict of Interest Statement

The authors declare that the research was conducted in the absence of any commercial or financial relationships that could be construed as a potential conflict of interest.
