# Supplementary material for: Polyploidy Index and Its Implications for the Evolution of Polyploids
Source: Front Genet. 2019 Sep 10;10:807. doi: 10.3389/fgene.2019.00807 (PMC6746930; doi:10.3389/fgene.2019.00807)
Supplement: Supplementary file 1 [file Table_1.docx]

**Supplementary Table 1 Information for original data material**

| **Order** | **Species name** | **Common name** | **Version** | **Accession** | **Data source** | **Journal** | **Reference** |
| --- | --- | --- | --- | --- | --- | --- | --- |
| 1 | *Vitis vinifera* | Grape vine | Genoscope.12X | CU459218–CU462737 | JGI (https://phytozome.jgi.doe.gov/pz/portal.html) | Nature | ([Jaillon et al., 2007](#_ENREF_16)) |
| 2 | *Populus trichocarpa* | Western poplar | v3.0 | AARH00000000 | JGI (https://phytozome.jgi.doe.gov/pz/portal.html) | Science | ([Tuskan et al., 2006](#_ENREF_34)) |
| 3 | *Arabidopsis thaliana* | Thale cress | TAIR10 |  | JGI (https://phytozome.jgi.doe.gov/pz/portal.html) | Nature | ([Initiative, 2000](#_ENREF_11)) |
| 4 | *Arabidopsis lyrata* | Lyrate rockcress | v1.0 |  | JGI (https://phytozome.jgi.doe.gov/pz/portal.html) | Nature Genetics | ([Hu et al., 2011](#_ENREF_8)) |
| 5 | *Brassica rapa* | Chinese cabbage | v1.3 |  | PGDD (http://chibba.agtec.uga.edu/duplication) | Nature Genetics | ([Wang et al., 2011](#_ENREF_40)) |
| 6 | *Brassica napus* | Oilseed rape | v1.0 | ERP005275/PRJEB6069 | *Brassica napus* Genome Resources  (http://www.genoscope.cns.fr/brassicanapus) | Science | ([Chalhoub et al., 2014](#_ENREF_2)) |
| 7 | *Brassica oleracea* | Cabbage | v1.5 |  | BRAD (http://brassicadb.org/brad/index.php) | Nature Communications | ([Liu et al., 2014](#_ENREF_23)) |
| 8 | *Raphanus sativus L.* | Radish | v1.0 |  | RGD (http://radish.kazusa.or.jp/) | DNA Research | ([Kitashiba et al., 2014](#_ENREF_20)) |
| 9 | *Theobroma cacao* | Cocoa bean | v1.1 | ALXC0100000 | JGI (https://phytozome.jgi.doe.gov/pz/portal.html) | Genome Biology | ([Motamayor et al., 2013](#_ENREF_26)) |
| 10 | *Gossypium arboreum* | Tree cotton | v1.0 | SRA150181 | Cotton gen (www.cottongen.org/) | Nature Genetics | ([Li et al., 2014](#_ENREF_21)) |
| 11 | *Gossypium hirsutum* | Upland cotton | v1.1 | PRJNA248163 | Cotton gen (www.cottongen.org/) | Nature Biotechnology | ([Zhang et al., 2015](#_ENREF_46)) |
| 12 | *Gossypium raimondii* | Cotton | v2.1 | PRJNA171262 | JGI (https://phytozome.jgi.doe.gov/pz/portal.html) | Nature | ([Paterson et al., 2012](#_ENREF_28)) |
| 13 | *Prunus persica* | Peach | v2.1 | AKXU02000000 | JGI (https://phytozome.jgi.doe.gov/pz/portal.html) | Nature Genetics | ([International Peach Genome et al., 2013](#_ENREF_13)) |
| 14 | *Prunus mume* | Mei | v1.0 |  | PGDD (http://chibba.agtec.uga.edu/duplication) | Nature Communications | ([Zhang et al., 2012b](#_ENREF_45)) |
| 15 | *Malus x domestica* | Apple | v1.0 |  | PGDD (http://chibba.agtec.uga.edu/duplication) | Nature Genetics | ([Velasco et al., 2010](#_ENREF_37)) |
| 16 | *Pyrus bretschneideri* | Pear | v1.0 |  | Pear Genome Project(http://peargenome.njau.edu.cn/) | Genome Research | ([Wu et al., 2013](#_ENREF_41)) |
| 17 | *Arachis duranensis* | Peanut | v1.0 | GCA_000817695.1 | Peanut Base (http://peanutbase.org/) | Nature Genetics | ([Bertioli et al., 2016](#_ENREF_1)) |
| 18 | *Arachis ipaensis* | Peanut | v1.0 | GCA_000816755.1. | Peanut Base (http://peanutbase.org/) | Nature Genetics | ([Bertioli et al., 2016](#_ENREF_1)) |
| 19 | *Lotus japonicus* | Lotus | v2.5 |  | PGDD (http://chibba.agtec.uga.edu/duplication) | DNA Research | ([Sato et al., 2008](#_ENREF_29)) |
| 20 | *Cicer arietinum* | Chickpea | v1.0 | PRJNA175619 | PGDD (http://chibba.agtec.uga.edu/duplication) | Nature Biotechnology | ([Varshney et al., 2013](#_ENREF_36)) |
| 21 | *Medicago truncatula* | Barrel medic | Mt4.0v1 | GCA000219495.2 | JGI (https://phytozome.jgi.doe.gov/pz/portal.html) | Nature | ([Young et al., 2011](#_ENREF_43)) |
| 22 | *Cajanus cajan* | Pigeonpea | Nov 2011 |  | PGDD (http://chibba.agtec.uga.edu/duplication) | Nature Biotechnology | ([Varshney et al., 2012](#_ENREF_35)) |
| 23 | *Glycine max* | Soybean | Wm82.a2.v1 | ACUP00000000 | JGI (https://phytozome.jgi.doe.gov/pz/portal.html) | Nature | ([Schmutz et al., 2010](#_ENREF_30)) |
| 24 | *Vigna angularis* | Adzuki bean | adzuki_ver3 |  | Crop Genomics Lab.(http://plantgenomics.snu.ac.kr/) | Proceedings of the National Academy of Sciences | ([Yang et al., 2015](#_ENREF_42)) |
| 25 | *Vigna radiata* | Mung bean | Vradi.ver6 |  | Crop Genomics Lab.(http://plantgenomics.snu.ac.kr/) | Nature Communications | ([Kang et al., 2014](#_ENREF_18)) |
| 26 | *Phaseolus vulgaris* | Common bean | v1.0 | PRJNA221782 | JGI (https://phytozome.jgi.doe.gov/pz/portal.html) | Genome Biology | ([Vlasova et al., 2016](#_ENREF_38)) |
| 27 | *Coffea canephora* | Coffee | v1.0 |  | Coffee Genome Hub (http://coffee-genome.org/) | Science | ([Denoeud et al., 2014](#_ENREF_4)) |
| 28 | *Capsicum annuum* | Pepper | CM334 V1.55 | AYRZ00000000 | Pepper Genome Platform  (http://peppergenome.snu.ac.kr/) | Nature Genetics | ([Kim et al., 2014](#_ENREF_19)) |
| 29 | *Solanum melongena* L. | Eggplant | SME_r2.5.1 | DRA001153 | Eggplant Genome Database  (http://eggplant.kazusa.or.jp/) | DNA Research | ([Hirakawa et al., 2014](#_ENREF_7)) |
| 30 | *Solanum tuberosum* L. | Potato | PGSC V3.4 |  | JGI (https://phytozome.jgi.doe.gov/pz/portal.html) | Nature Genetics | ([Huang et al., 2009b](#_ENREF_10)) |
| 31 | *Solanum lycopersicum* | Tomato | iTAG2.3 |  | JGI (https://phytozome.jgi.doe.gov/pz/portal.html) | Nature | ([Tomato Genome, 2012](#_ENREF_33)) |
| 32 | *Spirodela polyrhiza* | Greater duckweed | v2.0 |  | JGI (https://phytozome.jgi.doe.gov/pz/portal.html) | Nature Communications | ([Wang et al., 2014](#_ENREF_39)) |
| 33 | *Musa acuminata* | Banana | v1.0 |  | JGI (https://phytozome.jgi.doe.gov/pz/portal.html) | Nature | ([D'Hont et al., 2012](#_ENREF_3)) |
| 34 | *Elaeis guineensis* | Oil palm | v2.0 | ASJS00000000 | PGDD (http://chibba.agtec.uga.edu/duplication) | Nature | ([Singh et al., 2013](#_ENREF_32)) |
| 35 | *Oryza sativa* | Rice | v7_JGI |  | JGI (https://phytozome.jgi.doe.gov/pz/portal.html) | Nature | ([International Rice Genome Sequencing, 2005](#_ENREF_14)) |
| 36 | *Aegilops tauschii* | Goat grass  (Wheat D) | ASM34733v1 | AOCO010000000 | Ensembl Plants (http://plants.ensembl.org/index.html) | Nature | ([Jia et al., 2013](#_ENREF_17)) |
| 37 | *Triticum aestivum* | Common wheat | v2.2 |  | URGI (https://wheat-urgi.versailles.inra.fr/) | Science | ([International Wheat Genome Sequencing, 2014](#_ENREF_15)) |
| 38 | *Triticum urartu* | Wheat A | v1.0 | AOTI01000000 | PGDD (http://chibba.agtec.uga.edu/duplication) | Nature | ([Ling et al., 2013](#_ENREF_22)) |
| 39 | *Brachypodium distachyon* | Purple false brome | v3.1 | ADDN01000000 | JGI (https://phytozome.jgi.doe.gov/pz/portal.html) | Nature | ([International Brachypodium, 2010](#_ENREF_12)) |
| 40 | *Setaria italica* | Foxtail millet | v2.2 | AGNK01000000 | JGI (https://phytozome.jgi.doe.gov/pz/portal.html) | Nature Biotechnology | ([Zhang et al., 2012a](#_ENREF_44)) |
| 41 | *Sorghum bicolor* | Cereal grass | v3.1 | ABXC03000000 | JGI (https://phytozome.jgi.doe.gov/pz/portal.html) | Nature | ([Paterson et al., 2009](#_ENREF_27)) |
| 42 | *Zea mays* | Maize | Ensembl-18 |  | JGI (https://phytozome.jgi.doe.gov/pz/portal.html) | Science | ([Schnable et al., 2009](#_ENREF_31)) |
| 43 | *Citrullus lanatus* | Watermelon | v1.0 |  | Cucurbit Genomics Database (http://www.icugi.org/) | Nature Genetics | ([Guo et al., 2013](#_ENREF_6)) |
| 44 | *Cucumis sativus L.* | Cucumber | v1.0 |  | Cucurbit Genomics Database (http://www.icugi.org/) | Nature Genetics | ([Huang et al., 2009a](#_ENREF_9)) |
| 45 | *Cucumis melo L.* | Melon | v1.0 | ERP001463 | MELONOMICS (https://melonomics.net/) | Proceedings of the National Academy of Sciences | ([Garciamas et al., 2012](#_ENREF_5)) |
| 46 | *Ananas comosus* | Pineapple | v3 |  | JGI (https://phytozome.jgi.doe.gov/pz/portal.html) | Nature genetics | ([Ming et al., 2015](#_ENREF_25)) |
| 47 | *Hordeum vulgare L.* | barely | R1 |  | JGI (https://phytozome.jgi.doe.gov/pz/portal.html) | Nature | ([Mascher et al., 2017](#_ENREF_24)) |

**References**

Bertioli, D.J., Cannon, S.B., Froenicke, L., Huang, G., Farmer, A.D., Cannon, E.K., et al. (2016). The genome sequences of Arachis duranensis and Arachis ipaensis, the diploid ancestors of cultivated peanut. *Nat Genet* 48(4)**,** 438-446. doi: 10.1038/ng.3517.

Chalhoub, B., Denoeud, F., Liu, S., Parkin, I.A.P., Tang, H., Wang, X., et al. (2014). Early allopolyploid evolution in the post-Neolithic Brassica napus oilseed genome. *Science* 345(345)**,** 950-953.

D'Hont, A., Denoeud, F., Aury, J.M., Baurens, F.C., Carreel, F., Garsmeur, O., et al. (2012). The banana (Musa acuminata) genome and the evolution of monocotyledonous plants. *Nature* 488(7410)**,** 213-217. doi: 10.1038/nature11241.

Denoeud, F., Carretero-Paulet, L., Dereeper, A., Droc, G., Guyot, R., Pietrella, M., et al. (2014). The coffee genome provides insight into the convergent evolution of caffeine biosynthesis. *Science* 345(6201)**,** 1181-1184. doi: 10.1126/science.1255274.

Garciamas, J., Benjak, A., Sanseverino, W., Bourgeois, M., Mir, G., González, V.M., et al. (2012). The genome of melon (Cucumis melo L.). *Proceedings of the National Academy of Sciences of the United States of America* 109(29)**,** 11872.

Guo, S., Zhang, J., Sun, H., Salse, J., Lucas, W.J., Zhang, H., et al. (2013). The draft genome of watermelon (Citrullus lanatus) and resequencing of 20 diverse accessions. *Nature Genetics* 45(1)**,** 51-U82.

Hirakawa, H., Shirasawa, K., Miyatake, K., Nunome, T., Negoro, S., Ohyama, A., et al. (2014). Draft genome sequence of eggplant (Solanum melongena L.): the representative solanum species indigenous to the old world. *DNA Res* 21(6)**,** 649-660. doi: 10.1093/dnares/dsu027.

Hu, T.T., Pattyn, P., Bakker, E.G., Cao, J., Cheng, J.F., Clark, R.M., et al. (2011). The Arabidopsis lyrata genome sequence and the basis of rapid genome size change. *Nat Genet* 43(5)**,** 476-481. doi: 10.1038/ng.807.

Huang, S., Li, R., Zhang, Z., Li, L., Gu, X., Fan, W., et al. (2009a). The genome of the cucumber, Cucumis sativus L. *Nature Genetics* 41(12)**,** 1275-1281.

Huang, S., Li, R., Zhang, Z., Li, L., Gu, X., Fan, W., et al. (2009b). The genome of the cucumber, Cucumis sativus L. *Nat Genet* 41(12)**,** 1275-1281. doi: 10.1038/ng.475.

Initiative, A.G. (2000). Analysis of the genome sequence of the flowering plant Arabidopsis thaliana. *Nature* 408(6814)**,** 796-815.

International Brachypodium, I. (2010). Genome sequencing and analysis of the model grass Brachypodium distachyon. *Nature* 463(7282)**,** 763-768. doi: 10.1038/nature08747.

International Peach Genome, I., Verde, I., Abbott, A.G., Scalabrin, S., Jung, S., Shu, S., et al. (2013). The high-quality draft genome of peach (Prunus persica) identifies unique patterns of genetic diversity, domestication and genome evolution. *Nat Genet* 45(5)**,** 487-494. doi: 10.1038/ng.2586.

International Rice Genome Sequencing, P. (2005). The map-based sequence of the rice genome. *Nature* 436(7052)**,** 793-800. doi: 10.1038/nature03895.

International Wheat Genome Sequencing, C. (2014). A chromosome-based draft sequence of the hexaploid bread wheat (Triticum aestivum) genome. *Science* 345(6194)**,** 1251788. doi: 10.1126/science.1251788.

Jaillon, O., Aury, J.M., Noel, B., Policriti, A., Clepet, C., Casagrande, A., et al. (2007). The grapevine genome sequence suggests ancestral hexaploidization in major angiosperm phyla. *Nature* 449(7161)**,** 463-467. doi: 10.1038/nature06148.

Jia, J., Zhao, S., Kong, X., Li, Y., Zhao, G., He, W., et al. (2013). Aegilops tauschii draft genome sequence reveals a gene repertoire for wheat adaptation. *Nature* 496(7443)**,** 91-95. doi: 10.1038/nature12028.

Kang, Y.J., Kim, S.K., Kim, M.Y., Lestari, P., Kim, K.H., Ha, B.K., et al. (2014). Genome sequence of mungbean and insights into evolution within Vigna species. *Nat Commun* 5**,** 5443. doi: 10.1038/ncomms6443.

Kim, S., Park, M., Yeom, S.I., Kim, Y.M., Lee, J.M., Lee, H.A., et al. (2014). Genome sequence of the hot pepper provides insights into the evolution of pungency in Capsicum species. *Nat Genet* 46(3)**,** 270-278. doi: 10.1038/ng.2877.

Kitashiba, H., Li, F., Hirakawa, H., Kawanabe, T., Zou, Z., Hasegawa, Y., et al. (2014). Draft sequences of the radish (Raphanus sativus L.) genome. *DNA Res* 21(5)**,** 481-490. doi: 10.1093/dnares/dsu014.

Li, F., Fan, G., Wang, K., Sun, F., Yuan, Y., Song, G., et al. (2014). Genome sequence of the cultivated cotton Gossypium arboreum. *Nat Genet* 46(6)**,** 567-572. doi: 10.1038/ng.2987.

Ling, H.Q., Zhao, S., Liu, D., Wang, J., Sun, H., Zhang, C., et al. (2013). Draft genome of the wheat A-genome progenitor Triticum urartu. *Nature* 496(7443)**,** 87-90. doi: 10.1038/nature11997.

Liu, S., Liu, Y., Yang, X., Tong, C., Edwards, D., Parkin, I.A., et al. (2014). The Brassica oleracea genome reveals the asymmetrical evolution of polyploid genomes. *Nat Commun* 5**,** 3930. doi: 10.1038/ncomms4930.

Mascher, M., Gundlach, H., Himmelbach, A., Beier, S., Twardziok, S.O., Wicker, T., et al. (2017). A chromosome conformation capture ordered sequence of the barley genome. *Nature* 544(7651)**,** 427-433. doi: 10.1038/nature22043.

Ming, R., VanBuren, R., Wai, C.M., Tang, H., Schatz, M.C., Bowers, J.E., et al. (2015). The pineapple genome and the evolution of CAM photosynthesis. *Nat Genet* 47(12)**,** 1435-1442. doi: 10.1038/ng.3435.

Motamayor, J.C., Mockaitis, K., Schmutz, J., Haiminen, N., Livingstone, D., 3rd, Cornejo, O., et al. (2013). The genome sequence of the most widely cultivated cacao type and its use to identify candidate genes regulating pod color. *Genome Biol* 14(6)**,** r53. doi: 10.1186/gb-2013-14-6-r53.

Paterson, A.H., Bowers, J.E., Bruggmann, R., Dubchak, I., Grimwood, J., Gundlach, H., et al. (2009). The Sorghum bicolor genome and the diversification of grasses. *Nature* 457(7229)**,** 551-556. doi: 10.1038/nature07723.

Paterson, A.H., Wendel, J.F., Gundlach, H., Guo, H., Jenkins, J., Jin, D., et al. (2012). Repeated polyploidization of Gossypium genomes and the evolution of spinnable cotton fibres. *Nature* 492(7429)**,** 423-427. doi: 10.1038/nature11798.

Sato, S., Nakamura, Y., Kaneko, T., Asamizu, E., Kato, T., Nakao, M., et al. (2008). Genome structure of the legume, Lotus japonicus. *DNA Res* 15(4)**,** 227-239. doi: 10.1093/dnares/dsn008.

Schmutz, J., Cannon, S.B., Schlueter, J., Ma, J., Mitros, T., Nelson, W., et al. (2010). Genome sequence of the palaeopolyploid soybean. *Nature* 463(7278)**,** 178-183. doi: 10.1038/nature08670.

Schnable, P.S., Ware, D., Fulton, R.S., Stein, J.C., Wei, F., Pasternak, S., et al. (2009). The B73 maize genome: complexity, diversity, and dynamics. *Science* 326(5956)**,** 1112-1115. doi: 10.1126/science.1178534.

Singh, R., Ong-Abdullah, M., Low, E.T., Manaf, M.A., Rosli, R., Nookiah, R., et al. (2013). Oil palm genome sequence reveals divergence of interfertile species in Old and New worlds. *Nature* 500(7462)**,** 335-339. doi: 10.1038/nature12309.

Tomato Genome, C. (2012). The tomato genome sequence provides insights into fleshy fruit evolution. *Nature* 485(7400)**,** 635-641. doi: 10.1038/nature11119.

Tuskan, G.A., Difazio, S., Jansson, S., Bohlmann, J., Grigoriev, I., Hellsten, U., et al. (2006). The genome of black cottonwood, Populus trichocarpa (Torr. & Gray). *Science* 313(5793)**,** 1596-1604. doi: 10.1126/science.1128691.

Varshney, R.K., Chen, W., Li, Y., Bharti, A.K., Saxena, R.K., Schlueter, J.A., et al. (2012). Draft genome sequence of pigeonpea (Cajanus cajan), an orphan legume crop of resource-poor farmers. *Nat Biotechnol* 30(1)**,** 83-89. doi: 10.1038/nbt.2022.

Varshney, R.K., Song, C., Saxena, R.K., Azam, S., Yu, S., Sharpe, A.G., et al. (2013). Draft genome sequence of chickpea (Cicer arietinum) provides a resource for trait improvement. *Nat Biotechnol* 31(3)**,** 240-246. doi: 10.1038/nbt.2491.

Velasco, R., Zharkikh, A., Affourtit, J., Dhingra, A., Cestaro, A., Kalyanaraman, A., et al. (2010). The genome of the domesticated apple (Malus x domestica Borkh.). *Nat Genet* 42(10)**,** 833-839. doi: 10.1038/ng.654.

Vlasova, A., Capella-Gutierrez, S., Rendon-Anaya, M., Hernandez-Onate, M., Minoche, A.E., Erb, I., et al. (2016). Genome and transcriptome analysis of the Mesoamerican common bean and the role of gene duplications in establishing tissue and temporal specialization of genes. *Genome Biol* 17**,** 32. doi: 10.1186/s13059-016-0883-6.

Wang, W., Haberer, G., Gundlach, H., Gläßer, C., Nussbaumer, T., Luo, M.C., et al. (2014). The Spirodela polyrhiza genome reveals insights into its neotenous reduction fast growth and aquatic lifestyle. *Nature Communications* 5. doi: 10.1038/ncomms4311.

Wang, X., Wang, H., Wang, J., Sun, R., Wu, J., Liu, S., et al. (2011). The genome of the mesopolyploid crop species Brassica rapa. *Nat Genet* 43(10)**,** 1035-1039. doi: 10.1038/ng.919.

Wu, J., Wang, Z., Shi, Z., Zhang, S., Ming, R., Zhu, S., et al. (2013). The genome of the pear (Pyrus bretschneideri Rehd.). *Genome Res* 23(2)**,** 396-408. doi: 10.1101/gr.144311.112.

Yang, K., Tian, Z., Chen, C., Luo, L., Zhao, B., Wang, Z., et al. (2015). Genome sequencing of adzuki bean (Vigna angularis) provides insight into high starch and low fat accumulation and domestication. *Proc Natl Acad Sci U S A* 112(43)**,** 13213-13218. doi: 10.1073/pnas.1420949112.

Young, N.D., Debelle, F., Oldroyd, G.E., Geurts, R., Cannon, S.B., Udvardi, M.K., et al. (2011). The Medicago genome provides insight into the evolution of rhizobial symbioses. *Nature* 480(7378)**,** 520-524. doi: 10.1038/nature10625.

Zhang, G., Liu, X., Quan, Z., Cheng, S., Xu, X., Pan, S., et al. (2012a). Genome sequence of foxtail millet (Setaria italica) provides insights into grass evolution and biofuel potential. *Nat Biotechnol* 30(6)**,** 549-554. doi: 10.1038/nbt.2195.

Zhang, Q., Chen, W., Sun, L., Zhao, F., Huang, B., Yang, W., et al. (2012b). The genome of Prunus mume. *Nat Commun* 3**,** 1318. doi: 10.1038/ncomms2290.

Zhang, T., Hu, Y., Jiang, W., Fang, L., Guan, X., Chen, J., et al. (2015). Sequencing of allotetraploid cotton (Gossypium hirsutum L. acc. TM-1) provides a resource for fiber improvement. *Nat Biotechnol* 33(5)**,** 531-537. doi: 10.1038/nbt.3207.
